# SUMOylation of G9a regulates its function as an activator of myoblast proliferation

**DOI:** 10.1038/s41419-019-1465-9

**Published:** 2019-03-13

**Authors:** Shruti Srinivasan, Shilpa Rani Shankar, Yaju Wang, Reshma Taneja

**Affiliations:** Department of Physiology, Yong Loo Lin School of Medicine, National University of Singapore, 117593 Singapore, Singapore

## Abstract

The lysine methyltransferase G9a plays a role in many cellular processes. It is a potent repressor of gene expression, a function attributed to its ability to methylate histone and non-histone proteins. Paradoxically, in some instances, G9a can activate gene expression. However, regulators of G9a expression and activity are poorly understood. In this study, we report that endogenous G9a is SUMOylated in proliferating skeletal myoblasts. There are four potential SUMOylation consensus motifs in G9a. Mutation of all four acceptor lysine residues [K79, K152, K256, and K799] inhibits SUMOylation. Interestingly, SUMOylation does not impact G9a-mediated repression of MyoD transcriptional activity or myogenic differentiation. In contrast, SUMO-defective G9a is unable to enhance proliferation of myoblasts. Using complementation experiments, we show that the proliferation defect of primary myoblasts from conditional G9a-deficient mice is rescued by re-expression of wild-type, but not SUMOylation-defective, G9a. Mechanistically, SUMOylation acts as signal for PCAF (P300/CBP-associated factor) recruitment at E2F1-target genes. This results in increased histone H3 lysine 9 acetylation marks at E2F1-target gene promoters that are required for S-phase progression. Our studies provide evidence by which SUMO modification of G9a influences the chromatin environment to impact cell cycle progression.

## Introduction

Post-translational modifications (PTMs) such as acetylation, methylation, SUMOylation, ubiquitination, and phosphorylation rapidly and reversibly alter the function of cellular proteins. These modifications can promote or disrupt protein–protein interactions, permit or antagonize other modifications, and alter protein localization, stability, or conformation^1^.

SUMOylation is a conserved PTM that involves the covalent conjugation of small ubiquitin-like modifier (SUMO) protein to specific lysine residues in substrates. SUMOylation generally, although not exclusively, occurs at the consensus motif ψKxE/D, where ψ is a hydrophobic residue, K is the target lysine, x is any amino acid, followed by an acidic residue, although lysines that do not conform to the consensus are also modified^2,3^. The highly regulated SUMO modification is reversed by sentrin-specific proteases (SENPs)^4^. Growing evidence has shown that transcription factors and co-factors are key substrates for SUMOylation^5^. The covalent attachment of SUMO can alter subcellular localization of target proteins and their transcriptional activity. SUMOylation also serves as a signal for recruitment of proteins that contain a SUMO interaction motif (SIM)^6^. Histone modifiers that are recruited by SUMO-modified proteins regulate chromatin structure and transcription^7^. Through the diverse array of substrates that are modified, SUMOylation impacts many cellular processes including various phases of cell cycle progression^8–10^, cellular differentiation^11^, heterochromatin formation,^12^ and the DNA damage response^13^.

G9a and G9a-like protein (GLP) are SET-domain containing lysine methyltransferases that mono- and di-methylate histone 3 lysine 9 (H3K9me2) as well as several non-histone proteins to exert transcriptional silencing^14,15^. Both proteins are present in a complex and are required for global H3K9me2. Nevertheless, they function in a non-redundant manner as loss of either G9a or GLP ablates H3K9me2 and results in early embryonic lethality^16^. G9a is expressed in myoblasts and its expression declines upon the induction of differentiation. We and others have previously demonstrated that G9a inhibits skeletal myogenesis by repression of MyoD- and MEF2-dependent myogenic differentiation genes in a methyltransferase activity-dependent manner^17–21^. In addition to repression of differentiation genes, G9a also actively promotes myoblast proliferation in a methylation-independent manner^22^. This is mediated by the interaction of G9a with the E2F1/PCAF (P300/CBP-associated factor) complex, which results in the activation of E2F1-target genes required for S-phase progression. Interestingly, G9a preferentially interacts with the E2F1/PCAF-activating complexes at the G1/S phase of the cell cycle, and with MyoD at the G2/M phase^22^. Nonetheless, the mechanisms by which G9a is able to both repress expression of myogenic genes and activate proliferation genes in myoblasts are unclear. In muscle cells, SUMOylation represses the transcriptional activity of pro-myogenic factors of the MEF2 family^23,24^. Furthermore, SUMO modification of Pax7 is required to maintain myoblasts in an undifferentiated state^25^. These results suggest that SUMOylation is important to restrain differentiation of muscle cells. Similar to G9a levels, a reduction in the overall SUMOylation of SUMO1 and SUMO2/3 targets during differentiation is seen in myoblasts^26^. We therefore examined if SUMO modification of G9a enables it to function as an activator of E2F1-dependent gene expression.

In this study, we demonstrate that G9a is SUMOylated in skeletal myoblasts. Interestingly, SUMOylation of G9a is required for its ability to transcriptionally activate genes, but not for its repressive function. G9a-deficient primary myoblasts proliferate less efficiently compared to control cells. This proliferation defect is rescued by wild-type, but not SUMO-defective, G9a. Mechanistically, we show that SUMOylated G9a is recognized by the histone acetyltransferase PCAF, and promotes PCAF-E2F1 association. This results in PCAF-dependent activating chromatin marks at E2F1-target gene promoters, and consequently S-phase progression. Our studies not only unravel a novel mechanism by which G9a function is regulated, but provide fundamental insights by which PTMs of chromatin proteins influence the chromatin environment to impact gene expression.

## Results

### G9a is SUMO-modified in proliferating myoblasts

**Fig. 1:**
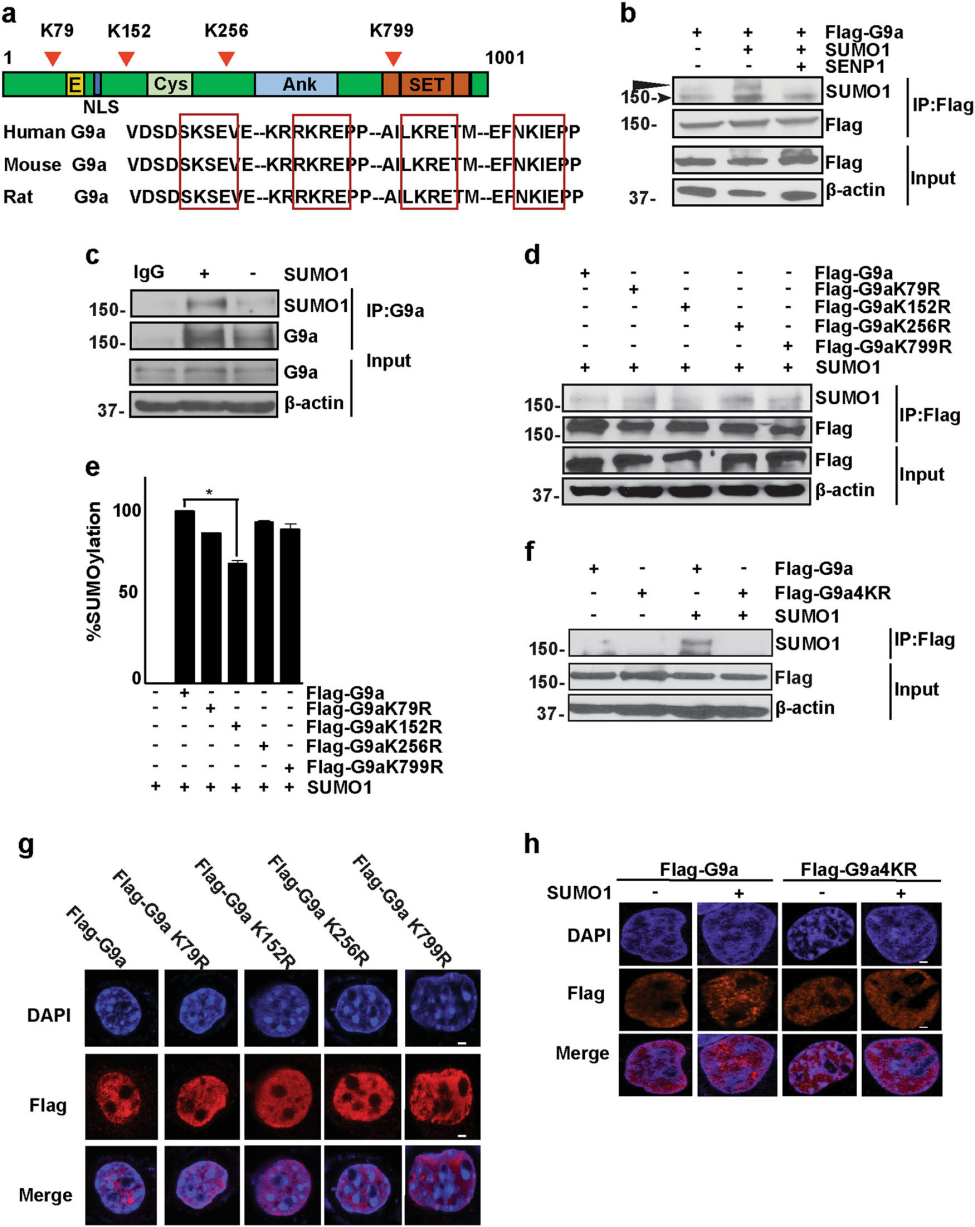
G9a is SUMO-modified. **a** Domain structure of G9a depicting the glutamine (E)- and cysteine (Cys)-rich regions, nuclear localization signal (NLS), ankyrin repeats (Ank), and the catalytic (SET) domain (upper panel). Numbers indicate amino acid residues. Arrowheads indicate potential sumoylation sites at lysine (K) 79 (K79), K152, K256, and K799. Alignment of the G9a complementary DNA (cDNA) sequence from humans (Gene ID: 10919), mouse (Gene ID: 110147), and rat (Gene ID: 361798) revealed a high degree of conservation of the putative SUMOylation sites (boxed). **b** HEK293T cells were transfected with constructs encoding Flag-G9a, SUMO1, and sentrin-specific protease 1 (SENP1) as indicated. Lysates were subjected to immunoprecipitation with Flag-agarose beads, followed by immunoblotting with anti-SUMO1 antibody. Anti-Flag antibody was used to detect the expression of G9a. β-Actin served as a loading control. Arrow indicates SUMOylated G9a. Arrowhead indicates a non-specific band. **c** C2C12 cells were transfected with pCS2 empty vector (−) or with a SUMO1 plasmid (+). Endogenous G9a was immunoprecipitated using anti-G9a antibody, followed by western blotting with anti-SUMO1 antibody. Immunoglobulin G (IgG) was used as a negative control. Ten percent of lysate was loaded to detect G9a expression (Input). β-Actin was used as a loading control. **d** HEK293T cells were transfected with constructs encoding wild-type Flag-G9a, and each of the single mutants, Flag-G9aK79R, Flag-G9aK152R, Flag-G9aK256R, Flag-G9aK799R, and SUMO1, as indicated. Lysates were immunoprecipitated with Flag-agarose beads, followed by western blotting with anti-SUMO1 antibody. **e** C2C12 myoblasts were transfected with Flag-G9a or the single mutants, Flag-G9aK79R, Flag-G9aK152R, Flag-G9aK2562R, and Flag-G9aK799R, in the presence of SUMO1. Nuclear extracts were used to perform a calorimetric global protein SUMOylation assay. Cells transfected with only SUMO1 were used as a negative control. Percentage SUMOylation for each mutant compared to wild-type G9a is shown. **f** Cells were transfected with Flag-G9a or Flag-G9a4KR in the absence or presence of SUMO1 as indicated. Lysates were immunoprecipitated with Flag-agarose beads, followed by western blotting with anti-SUMO1 antibody. **g** COS-7 cells were transfected with wild-type Flag-G9a and each of the single mutants, Flag-G9aK79R, Flag-G9aK152R, Flag-G9aK256R, and Flag-G9aK799R. Cells were fixed and stained with anti-Flag (red) antibody. Nuclei were stained and visualized with 4′,6-diamidino-2-phenylindole (DAPI) (blue). **h** COS-7 cells were transfected with Flag-G9a and Flag-G9a4KR in the absence or presence of SUMO1. Cells were fixed and stained with anti-Flag (red) antibody. Nuclei were stained and visualized with DAPI (blue). Scale bar = 100 μm. Significance was determined using Student’s t-test (*p<0.05)

In addition to its well-characterized role as a transcriptional co-repressor via its SET-domain-dependent H3K9me2 activity, a few recent studies have demonstrated that G9a functions as a transcriptional co-activator^22,27–32^. In muscle cells, we have previously demonstrated that G9a positively regulates expression of E2F1-target genes as part of the E2F1-PCAF complex. Nevertheless, signals that are required for the association of G9a in either repressive or co-activator complexes remain poorly understood. We hypothesized that PTMs may modulate association of G9a with distinct protein complexes. Analysis of the G9a protein sequence revealed four conserved SUMOylation consensus sites at lysine (K) 79 (K79), K152, K256, and K799 (Fig. 1a). To determine whether G9a undergoes SUMOylation, cells were co-transfected with Flag-G9a and SUMO1. Lysates were immunoprecipitated with Flag-agarose beads. Immunoblotting with anti-SUMO1 antibody showed that G9a is SUMO-modified (Fig. 1b). Co-transfection with SENP1, a SUMO-deconjugating enzyme, abrogated G9a SUMOylation. To detect SUMOylation in muscle cells, endogenous G9a was immunoprecipitated from proliferating C2C12 myoblasts transiently transfected with SUMO1. SUMOylated G9a band was significantly stronger in cells transfected with SUMO1 (Fig. 1c). We then examined whether K79, K152, K256, and K799 are bona fide SUMOylation sites. Lysine (K) to Arg (R) point mutants were generated using site-directed mutagenesis. Cells were co-transfected with wild-type G9a or each of the four single mutants and SUMO1. The single point mutants did not have a major impact on SUMOylation, suggesting compensatory mechanisms or simultaneous sumoylation at these sites (Fig. 1d). We further tested SUMOylation of the single mutants in proliferating myoblasts. Consistent with our previous results, Flag-G9aK79R, Flag-G9aK256R, and Flag-G9aK799R did not show a significant decrease in SUMOylation levels. Mutation of K152 to arginine decreased G9a SUMOylation by ~30% (Fig. 1e). On the other hand, mutation of all four lysine residues to arginine (G9a4KR) abrogated SUMOylation even in the presence of SUMO1 (Fig. 1f). Visualization by confocal microscopy also showed no difference in the subcellular localization of each single mutant protein (Fig. 1g), as well as G9a4KR mutant (Fig. 1h), suggesting that the nuclear localization of G9a is independent of its SUMOylation status.

### SUMOylation does not impact G9a-mediated repression of myogenic differentiation

**Fig. 2:**
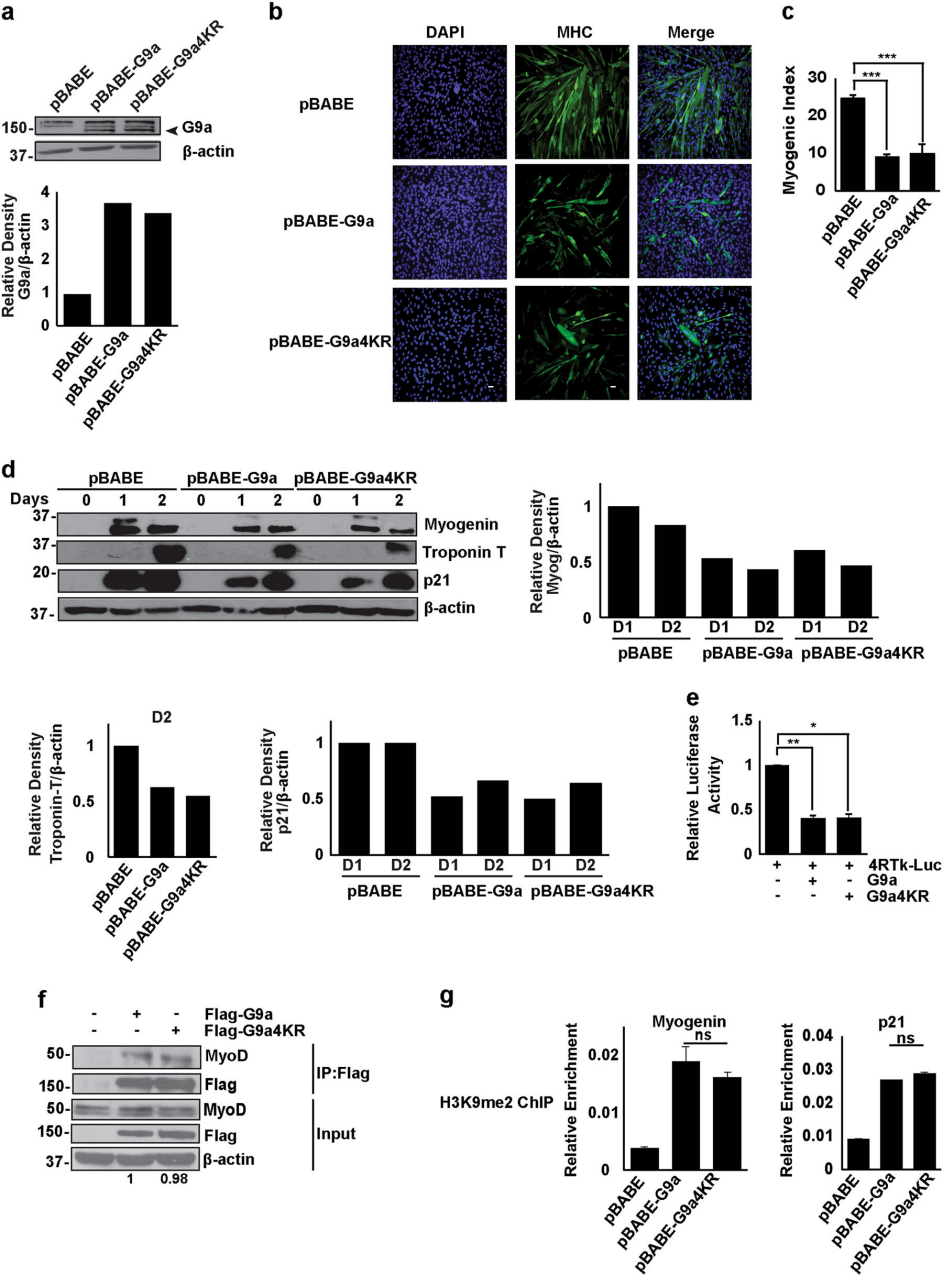
SUMOylation does not impact G9a-mediated repression of myogenic differentiation. **a** pBABE-G9a and pBABE-G9a4KR were retrovirally overexpressed in C2C12 cells. Control cells were infected with pBABE empty vector. G9a expression was determined by western blotting with anti-G9a antibody. Arrowhead indicates the expression of exogenous human G9a. The expression of G9a and G9a4KR was quantified by determining the band intensities relative to glyceraldehyde 3-phosphate dehydrogenase (GAPDH). The value in pBABE cells was given an arbitrary value of 1. G9a and G9a4KR were expressed ~3.7- and ~3.4-fold higher than control cells. **b**, **c** Major histocompatability complex (MHC) staining and myogenic index was assessed in pBABE-G9a- and pBABE-G9a4KR-expressing cells compared with control pBABE cells at day 2 of differentiation. **d** Cell lysates at days 0, 1, and 2 of differentiation from pBABE-, pBABE-G9a-, and pBABE-G9a4KR-expressing cells were analyzed by western blotting using Myogenin, Troponin T, and p21 antibodies. Bar graphs show quantification of the band intensities of Myogenin, Troponin T, and p21 at days 1 (D1) and D2 relative to β-actin. The values were plotted relative to pBABE, which was given a value of 1. **e** C2C12 cells stably expressing pBABE and pBABE-G9a or pBABE-G9a4KR were transfected with 4Rtk-Luc (200 ng) in triplicates. After 24 h, cells were harvested and luciferase activity was measured. **f** Flag-G9a and Flag-G9a4KR were transfected in C2C12 cells. G9a was immunoprecipitated using anti-Flag beads. Interaction with endogenous MyoD was analyzed by western blot. Endogenous expression of MyoD and exogenous expression of Flag was analyzed in lysates (Input). The band intensities of MyoD immunoprecipitation (IP) relative to Flag IP in the presence of G9a and G9a4KR were quantified. Numbers at the bottom of the panel show the band intensities normalized to G9a, which was given a value of 1. **g** H3K9me2 marks were determined at the promoters of Myogenin and p21 in cells stably expressing pBABE, pBABE-G9a, or pBABE-G9a4KR using chromatin immunoprecipitation. Error bars indicate mean ± S.D. Scale bar = 100 μm. NS not significant. Significance was determined using Student’s t-test (*p < 0.05, **p < 0.01, ***p < 0.001).

We and others previously reported the role of G9a as a transcriptional co-repressor in the inhibition of myogenic differentiation^17–22^. To understand the physiological relevance of G9a SUMOylation, we examined its impact on myogenic differentiation. C2C12 lines stably expressing wild-type G9a (pBABE-G9a) or the mutant G9a4KR (pBABE-G9a4KR) were generated. Expression was compared to control cells expressing the empty vector pBABE (Fig. 2a). Quantification of G9a levels compared to glyceraldehyde 3-phosphate dehydrogenase (GAPDH) revealed ~3.7- and 3.4-fold increase of G9a and G9a4KR in the stable cell lines. Stable overexpression of both G9a and G9a4KR resulted in significant inhibition of myogenic differentiation as evidenced by a reduced number of -myosin heavy chain-positive (MHC^+^) myotubes compared to the control cells (Fig. 2b, c). There was no apparent change in cell viability by overexpression of either G9a or G9a4KR. Consistent with the phenotype, cells stably expressing both G9a and G9a4KR exhibited reduced levels of myogenic differentiation markers Myogenin and Troponin T (Fig. 2d). Furthermore, both cell lines showed reduced expression of the cyclin-dependent kinase inhibitor p21, an important mediator of the cell cycle exit requisite for myogenic differentiation (Fig. 2d). Since G9a negatively regulates MyoD target genes involved in cell cycle exit and differentiation^22^, we transfected cells stably expressing G9a or G9a4KR with the myogenic regulatory factor (MRF) reporter 4Rtk-Luc^33^. The MRF reporter is robustly activated by MyoD and is a proxy for MyoD transcriptional activity. Consistent with previous reports^17,22^, overexpression of wild-type G9a significantly repressed reporter activity. Overexpression of G9a4KR also repressed the reporter activity (Fig. 2e). In addition, G9a and G9a4KR interacted similarly with endogenous MyoD in co-immunoprecipitation assays (Fig. 2f). The levels of the G9a signature H3K9me2-repressive marks on the promoters of two MyoD target genes Myogenin and p21 were comparably elevated in G9a and G9a4KR cells compared to the control cells (pBABE) (Fig. 2g). Together, these results indicate that G9a SUMOylation is dispensable for the methylation-dependent repression of differentiation.

### SUMOylation of G9a is required for its role in promoting myoblast proliferation

**Fig. 3:**
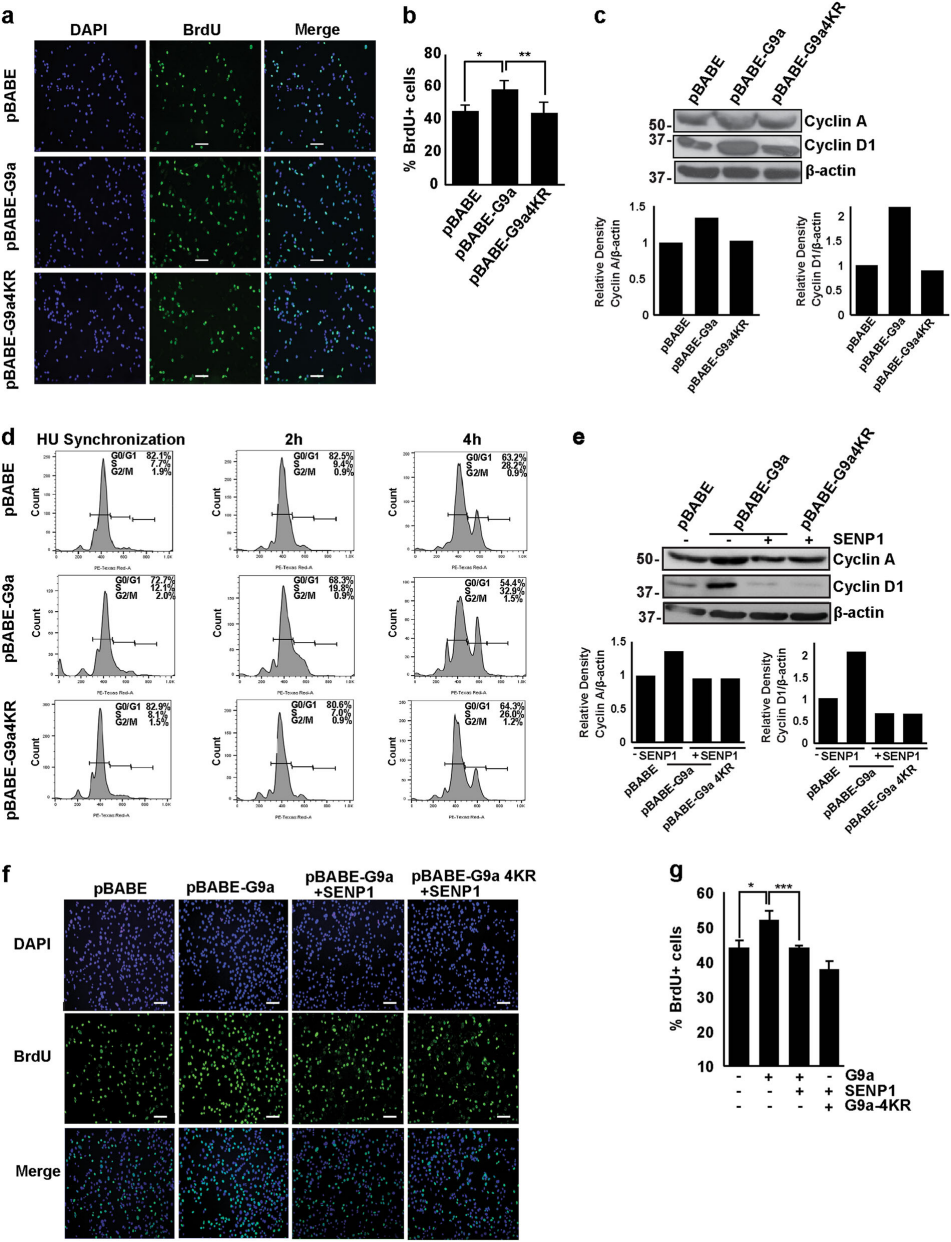
Mutation of SUMOylation sites abrogates G9a-mediated proliferation. **a**, **b** Cellular proliferation was measured by bromodeoxyuridine (BrdU) uptake in vector control, pBABE-G9a, and pBABE-G9a4KR cells. The percentage of BrdU^+^ cells is shown. **c** Lysates from proliferating pBABE, pBABE-G9a, and pBABE- G9a4KR cells were analyzed by western blotting using Cyclin D1, Cyclin A, and β-actin antibodies. The band intensities for Cyclin A and Cyclin D1 relative to β-actin were quantified in each cell line. The value in pBABE cells was given an arbitrary value of 1. **d** Flow cytometric analysis of pBABE-, pBABE-G9a-, and pBABE-G9a4KR-expressing cells synchronized at the G1/S boundary using hydroxyurea (HU). Cells were released into the growth media for 2 and 4 h before cell cycle analysis. Percentage of cells in G0/G1, S, and G2/M phases are indicated. **e** Lysates from proliferating pBABE-G9a and pBABE-G9a4KR cells transfected with pCS2 or SENP1 were analyzed by western blotting using Cyclin D1, Cyclin A, and β-actin antibodies. The expression of Cyclin A and Cyclin D1 was quantified relative to β-actin and is shown in the bar graph below. Control cells were given an arbitrary value of 1. **f**, **g** BrdU staining and percentage of BrdU^+^ cells was analyzed in proliferating pBABE, pBABE-G9a, and pBABE-G9a4KR cells transfected with either empty vector pCS2 or SENP1. Error bars indicate mean ± S.D. Scale bar = 100 μm. Significance was determined using Student’s t-test (*p < 0.05, **p< 0.01, ***p < 0.001).

To investigate the role of G9a SUMOylation in regulating proliferation of myoblasts, pBABE, pBABE-G9a, or pBABE-G9a4KR were pulsed with bromodeoxyuridine (BrdU) and stained with anti-BrdU antibody. Compared to control cells, pBABE-G9a cells exhibited an increase in BrdU incorporation. On the other hand, cells overexpressing G9a4KR did not show an increase in BrdU positivity compared to controls (Fig. 3a, b), suggesting that SUMOylation may impact G9a-dependent cellular proliferation. To confirm these findings, expression of the cell cycle genes Cyclin A and Cyclin D1, two important targets of the transcription factor E2F1, were examined. pBABE-G9a4KR showed lower levels of both proteins compared to cells overexpressing G9a (Fig. 3c). In addition, in cells synchronized at G1/S boundary using hydroxyurea (HU), a lower percentage of G9a4KR cells entered the S-phase upon release into growth media compared to G9a cells  at the time points analyzed (Fig. 3d). To validate the effect of SUMO modification on G9a-mediated increase in proliferation, pBABE-G9a and pBABE-G9a4KR cells were transfected with SENP1. Both Cyclin A and Cyclin D1 levels were blunted in response to SENP1 in pBABE-G9a cells, whereas G9a4KR cells were refractory (Fig. 3e). Correspondingly, the percentage of BrdU^+^ cells was reduced upon SENP1 expression compared to cells overexpressing G9a (Fig. 3f). Based on these results, we concluded that SUMOylation of G9a is critical for its role in promoting myoblast proliferation.

### SUMO modification of G9a regulates PCAF-E2F1 association

**Fig. 4:**
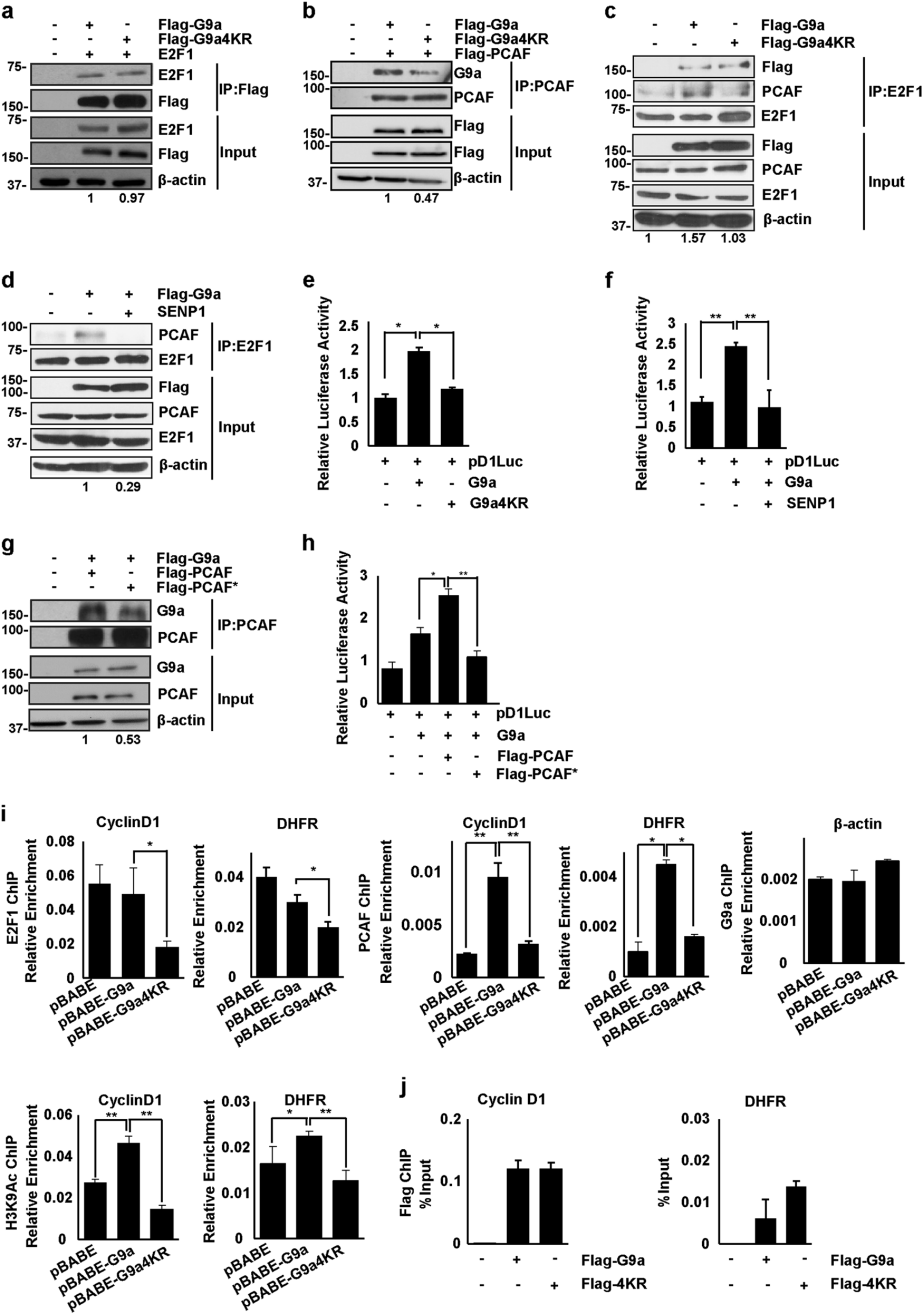
G9a SUMOylation regulates E2F1/PCAF (P300/CBP-associated factor) association. **a** HEK293T cells were co-transfected with E2F1 and Flag-G9a or Flag-G9a4KR. Lysates were immunoprecipitated with Flag-agarose beads and immunoblotted with anti-E2F1, anti-G9a, and anti-β-actin antibodies. Numbers at the bottom of the panel indicate the relative interaction of E2F1 with G9a and G9a4KR, which was quantified by calculating the ratio of E2F1 to Flag band intensities in the immunoprecipitated material. The interaction with G9a was given a value of 1. **b** HEK293T cells were co-transfected with PCAF and Flag-G9a or Flag-G9a4KR. Lysates were immunoprecipitated with anti-PCAF antibody and immunoblotted with anti-PCAF, anti-G9a, and anti-β-actin antibodies. The relative interaction of PCAF with G9a and G9a4KR was quantified by calculating the ratio of G9a to PCAF band intensities in the immunoprecipitated material. The interaction with G9a was given a value of 1. **c** C2C12 cells were transfected with Flag-G9a or Flag-G9a4KR. Lysates were immunoprecipitated with anti-E2F1 antibody and immunoblotted with anti-PCAF and anti-FLAG antibodies. The ratio of PCAF to E2F1 band intensities was quantified in the immunoprecipitated material. Numbers at the bottom of the panel show interaction relative to control cells which was given a value of 1. **d** Flag-G9a was transfected with or without SENP1 as indicated. Lysates were immunoprecipitated with anti-E2F1 antibody and immunoblotted with anti-PCAF, anti-E2F1, and anti-β-actin antibodies. The band intensities of PCAF with E2F1 were quantified in the immunoprecipitated material. The interaction in the presence of G9a was given a value of 1. **e** Cells stably expressing pBABE-G9a or pBABE-G9a4KR were transfected with 200 ng of Cyclin D1 promoter (pD1luc) reporter. After 48 h, the luciferase activity was measured using dual luciferase reporter assays. **f** pBABE and pBABE-G9a cells were transfected with 200 ng of Cyclin D1 promoter (pD1luc) with or without SENP1. Luciferase activity was measured using dual luciferase reporter assays. **g** HEK293T cells were co-transfected with Flag-G9a and PCAF or PCAF-SUMO interaction motif (SIM) mutant (PCAF*). Lysates were immunoprecipitated with anti-PCAF antibody, followed by immunoblotting with anti-PCAF, anti-G9a, and anti- β-actin antibodies. The interaction of PCAF and PCAF* with G9a was quantified by calculating the ratio of G9a to PCAF band intensities in the immunoprecipitated material. Numbers at the bottom of the panel indicate interaction relative to PCAF that was given a value of 1. **h** pBABE and pBABE-G9a cells were co-transfected with 200 ng of Cyclin D1 reporter along with PCAF or PCAF SIM mutant (PCAF*). Luciferase activity was measured using dual luciferase reporter assays. **i** Chromatin immunoprecipitation (ChIP) assays were performed with anti-E2F1, anti-PCAF, and anti-H3K9ac antibodies at Cyclin D1 and dihydrofolate reductase (DHFR) promoters in proliferating pBABE, pBABE-G9a, or pBABE-G9a4KR cells. As a negative control, G9a ChIP was performed at the β-actin promoter in pBABE, pBABE-G9a, and pBABE-G9a4KR cells. **j** No change in G9a occupancy was seen in Flag-G9a and Flag-G9a4KR cells using anti-Flag antibody. .Significance was determined using Student’s t-test (*p < 0.05, **p < 0.001, ****p < 0.001).

We have previously shown that G9a interacts with E2F1 and PCAF and enhances PCAF/E2F1 association, which is important for E2F1 activity^22,34^. This in turn facilitates PCAF-mediated H3K9ac-activating marks at E2F1-target promoters^22^. We tested whether G9a SUMOylation impacts its interaction with the E2F1-PCAF complex. Cells were transfected with Flag-G9a or Flag-G9a4KR and E2F1. Lysates were immunoprecipitated with Flag-agarose beads and immunoblotted with anti-E2F1. Both G9a and G9a4KR interacted with E2F1 (Fig. 4a). On the other hand, immunoprecipitation of lysates with anti-PCAF antibody showed reduced interaction between PCAF with G9a4KR compared to G9a (Fig. 4b), indicating that SUMOylation of G9a is important for its association with PCAF. Consistently, in proliferating C2C12 myoblasts, G9a and G9a4KR interacted equally with E2F1 (Fig. 4c). However, the interaction between E2F1 and PCAF was lower in cells overexpressing Flag-G9a4KR. To further test if G9a SUMOylation is required for PCAF recruitment to E2F1, we co-transfected SENP1 along with Flag-G9a in C2C12 cells. SENP1 significantly reduced the interaction between E2F1 and PCAF in the presence of G9a (Fig. 4d). To determine the impact of G9a SUMOylation on E2F1 transcriptional activity, we performed luciferase assays with the Cyclin D1 promoter reporter (pD1Luc) in pBABE-G9a or pBABE-G9a4KR cells. The Cyclin D1 promoter activity was significantly lower in G9a4KR cells with respect to wild-type G9a (Fig. 4e). Additionally, SENP1 significantly reduced the Cyclin D1 promoter activity augmented by G9a (Fig. 4f). Together, these results suggested a model in which PCAF, through its SIM domain, is recognized by SUMOylated G9a, and thereby recruited to E2F1. We therefore mutated the hydrophobic amino acids valine (V), isoleucine (I), and leucine (L) in the SIM motif [Q**-IIV-**S**-L**] of PCAF. Interestingly, consistent with our prediction, the SIM mutant PCAF (PCAF*) showed reduced interaction with G9a (Fig. 4g). Moreover, unlike wild-type PCAF, PCAF* did not enhance Cyclin D1 reporter activity in pBABE-G9a cells (Fig. 4h). We next examined E2F1 and PCAF recruitment at the Cyclin D1 and dihydrofolate reductase (DHFR) promoters. In cells overexpressing pBABE-G9a4KR, we saw a significant reduction in the occupancy of E2F1 at both promoters compared to pBABE-G9a cells (Fig. 4i). Only cells overexpressing G9a showed enrichment of PCAF and H3K9Ac, a signature of PCAF activity at Cyclin D1 and DHFR promoters. Interestingly, G9a occupancy itself was not altered in G9a and G9a4KR cells (Fig. 4j). Together, these results demonstrate that the absence of G9a SUMOylation results in reduced PCAF recruitment and consequently activation marks at E2F1-target gene promoters. Since PCAF acetylates and enhances E2F1 DNA binding^34^, reduced PCAF recruitment may lead to a reduction of E2F1 occupancy.

### G9a SUMOylation is essential in promoting primary myoblast proliferation

**Fig. 5:**
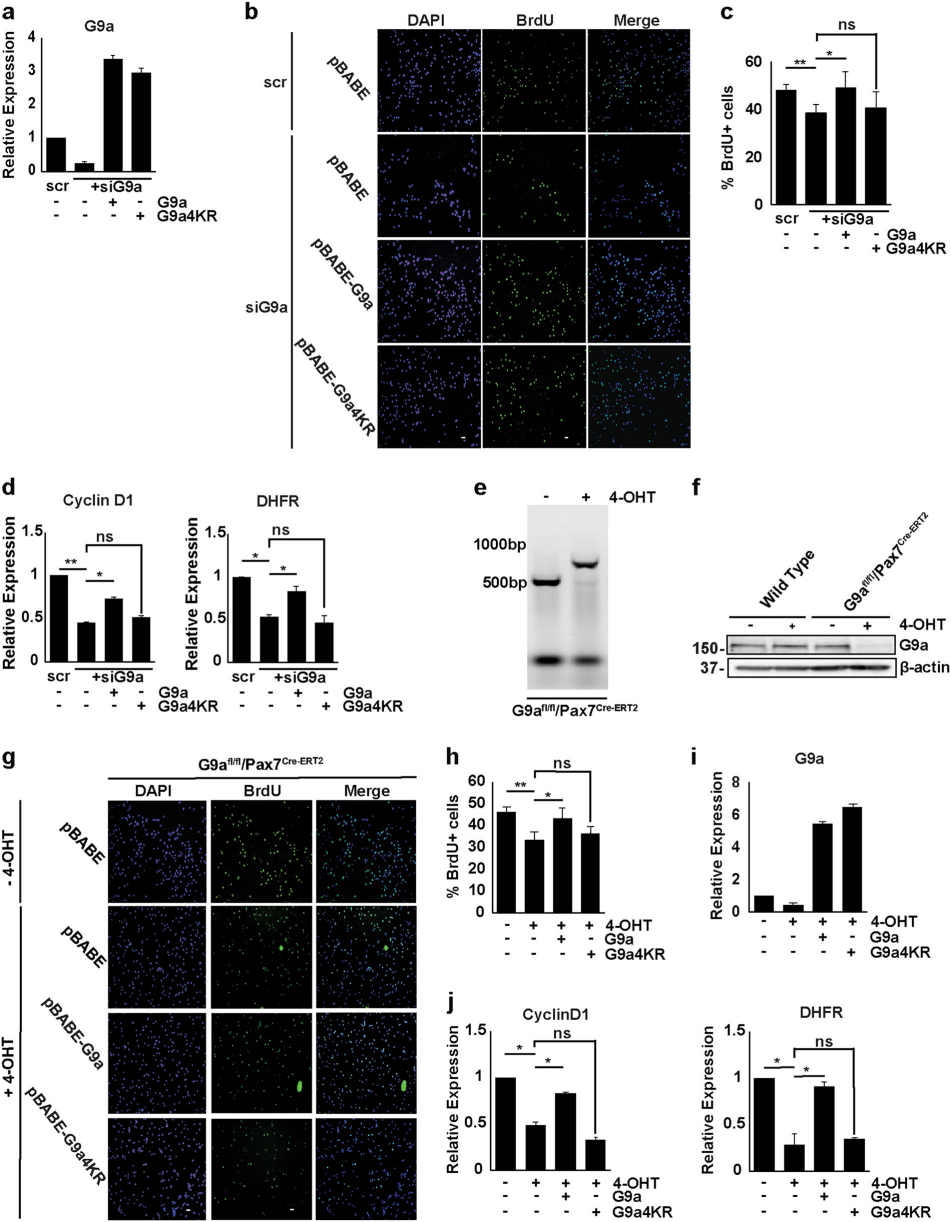
SUMOylation of G9a promotes myoblast proliferation. **a**, **b** Primary myoblasts isolated from wild-type mice were transduced with pBABE-G9a (human) and pBABE-G9a4KR retroviral expression vectors. Control cells were transfected with the pBABE vector. Endogenous mouse G9a was knocked down in all primary myoblast lines using small interfering RNA (siRNA) for 48 h. Control cells were transfected with scrambled siRNA. Cells were analyzed by quantitative real- time polymerase chain reaction (Q-PCR) for G9a expression. Cells were pulsed with bromodeoxyuridine (BrdU) and stained with anti-BrdU antibody. Nuclei were stained with 4′,6-diamidino-2-phenylindole (DAPI). **c** The percentage of BrdU^+^ cells is shown. **d** Expression of Cyclin D1 and dihydrofolate reductase (DHFR) was analyzed by Q-PCR. **e** Excision of floxed G9a allele was confirmed by PCR 48 h of 4-hydroxytamoxifen (4-OHT) treatment of primary myoblasts isolated from G9a^fl/fl^/Pax7^Cre-ERT2^ mice. **f** Primary myoblasts isolated from wild-type and G9a^fl/fl^/Pax7^Cre-ERT2^ mice were treated with 4-OHT for 48 h. G9a expression was assessed using western blot. **g**–**j** Human G9a and G9a4KR were retrovirally overexpressed in primary myoblasts isolated from G9a^fl/fl^/Pax7^Cre-ERT2^ mice. Control cells were transfected with the pBABE vector. Cells were subsequently treated with 4-OHT for 48 h. Cells were pulsed with BrdU and stained with anti-BrdU. The percentage of BrdU^+^ cells is shown in **h**. Expression of G9a (**i**), Cyclin D1, and DHFR (**j**) was analyzed by Q-PCR in control (untreated) and 4-OHT-treated cells (pBABE, pBABE-G9a, and pBABE-G9a4KR). Scale bar = 100 μm. NS not significant. Significance was determined using Student’s t-test (*p < 0.05, **p < 0.01)

To examine the relevance of SUMOylation in G9a-mediated myoblast proliferation ex vivo, we used two approaches. In the first approach, we established primary myoblasts from wild-type mice. Cells were transduced with human pBABE-G9a and pBABE-G9a4KR. After small interfering RNA (siRNA)-mediated knockdown of endogenous mouse G9a, we assessed their proliferative capacity and E2F1-target gene expression. Primary myoblasts transfected with scrambled siRNA (scr) were used as control. Knockdown of endogenous G9a reduced cell proliferation. Cells stably overexpressing pBABE-G9a showed increased BrdU incorporation compared to control cells transfected with scrsiRNA (Fig. 5a–c), whereas proliferation of cells expressing pBABE-G9a4KR was similar to siG9a cells. We also analyzed the expression of cell cycle markers regulated by G9a using quantitative real-time polymerase chain reaction (Q-PCR) (Fig. 5d). G9a, but not G9a4KR, was able to rescue the expression of E2F1-target genes compared to siG9a cells. In the second approach, proliferation of G9a^fl/fl^/Pax7^CreERT2^ primary myoblasts was examined ex vivo. Conditional excision of the G9a allele upon treatment with 4-hydroxytamoxifen (4-OHT) was confirmed by PCR (Fig. 5e), and loss of G9a protein was confirmed by western blot (Fig. 5f). We then stably expressed pBABE, pBABE-G9a, and pBABE-G9a4KR in G9a-null myoblasts and compared their proliferation. Upon 4-OHT treatment, proliferation of primary myoblasts was reduced. Interestingly, re-expression of wild-type G9a, but not of G9a4KR, rescued proliferation (Fig. 5g–i) and expression of E2F1-target genes (Fig. 5j), confirming that SUMOylation of G9a is a determinant of its function as an activator of proliferation and E2F1-target gene expression.

## Discussion

In this study, we have uncovered a novel PTM that regulates G9a transcriptional activity and underlies its unconventional role as an activator of gene expression. We demonstrate that G9a undergoes SUMOylation in muscle precursor cells, which serves as a signal for recruitment of PCAF to the E2F1 complex. This results in transcriptional activation marks at the promoters of E2F1-dependent S-phase genes, and thereby enhances the proliferative capacity of myoblasts.

In addition to its well-established role in mediating transcriptional repression via its H3K9me2 activity, G9a has been reported to function as a transcriptional co-activator through several distinct mechanisms. Early studies showed that G9a binds to the estrogen receptor (ER) in a ligand-dependent manner to activate ER-target genes^29^. More recently, G9a was found to methylate ER, a signal recognized by PHF20/MOF histone acetyltransferase complex to result in activation marks at ER-target genes in breast cancer cells^31^. In the context of a subset of glucocorticoid receptor (GR)-activated genes, G9a functions as a scaffold to recruit co-activators CARM1 and p300^27,30^. PTMs have been found to regulate G9a transcriptional activity. Auto-methylation at K185 is important for association of G9a with GR and HP1γ. Phosphorylation at an adjacent threonine (T186) by aurora kinase B blocks this interaction, suggesting that PTMs switch G9a function as co-activator or co-repressor on GR-regulated genes^32^. Activation of the β^maj^ globin gene by G9a involves its association with Pol II and formation of the pre-initiation complex^28^. In most but not all of these studies, G9a-mediated activation of gene expression was found to be independent of its methyltransferase activity. Our findings provide evidence for SUMOylation as a regulator of G9a transcriptional activity. Our overall findings are also consistent with previous studies demonstrating that G9a−/− mouse embryonic fibroblasts (MEFs) proliferate slower than wild-type MEFs with reduced numbers in the S phase^35^, and with the established role of SUMOylation in cell cycle progression^8–10^.

A large number of studies have associated SUMOylation of proteins with transcriptional repression^5,7,36^. Surprisingly, however, chromatin immunoprecipitation (ChIP) assays using an antibody against the yeast SUMO peptide showed that SUMOylated proteins are associated with promoters of actively transcribed, but not repressed, genes in *Saccharomyces* *cerevisiae*^37^. Moreover, genome-wide studies have also identified SUMO to be present in active open chromatin regions^38^. Indeed, several studies have validated that SUMO-conjugation activates proteins including p53^39^, TCF4^40^, Ikaros^41^, FoxM1^42^, and Dnmt3a^43^. SUMOylation can result in transcriptional activation by blocking other PTMs, promoting or disrupting protein–protein interactions, and altering protein conformation. SUMOylation also serves as a signal for recruitment of chromatin-modifying complexes that change the activity of the SUMO-modified proteins. Our studies show that SUMOylation of G9a functions as a recruitment signal for PCAF. The SIM in PCAF at positions 128–133 allows its SUMO-dependent interaction with G9a.

Previous studies have shown that G9a inhibits myogenesis in primary myoblasts and cultured muscle cell lines^17–21^. In addition, conditional knockout of G9a in the brain results in the upregulation of myogenic genes, demonstrating that in vivo it functions as a global suppressor of myogenesis^44^. Compensatory mechanisms and redundancy with GLP might account for the lack of an overt phenotype in muscle development in vivo upon conditional ablation of G9a in the skeletal muscle^45^.

G9a is involved in several biological processes, yet regulators of its expression or activity are poorly understood. The overexpression of G9a has been reported in many different types of cancers and is associated with poor prognosis^14,46^. Consistent with the findings in this study, G9a expression is elevated in rhabdomyosarcoma, a skeletal muscle tumor that exhibits a block in muscle differentiation. Down-regulation of G9a expression or activity using pharmacological inhibitors reduces tumor growth and motility (unpublished data). Growing evidence indicates that the upregulation of components of the SUMO machinery in cancers is associated with increased proliferation, drug resistance, and cancer stem cell maintenance^47^. Our studies imply that targeting SUMOylation may provide an alternative strategy to G9a-based cancer therapies. In addition, it would be interesting to determine if G9a expression and/or its SUMOylation status is altered in muscular dystrophies as well as upon aging, where reduced proliferation of myoblasts is central to the loss of muscle regenerative potential.

## Materials and methods

### Primary myoblasts and cell culture

All animal protocols were approved by the Institutional Animal Care and Use Committee at the National University of Singapore. Conditional ablation of G9a in the muscle was achieved by crossing G9a^fl/fl^ mice^44^ with Pax7^CreERT2^ mice (Jackson Labs; strain 012476). Primary myoblasts were isolated from the hind limb muscles of 1–2-month-old wild-type and G9a^fl/fl^/Pax7^CreERT2^ mice as previously described^48,49^. Briefly, tissue was enzymatically digested and cells were plated on collagen (Sigma)-coated plates in F-10 media (Gibco) containing 20% fetal bovine serum (FBS) and 5 ng/ml basic fibroblast growth factor (Invitrogen). Myoblasts were enriched by pre-plating cells for 15–30 min after trypsinization for the first few passages. The purity of myoblasts was confirmed by staining with anti-Pax7 antibody. Cultures having >95% Pax7 positivity were used. To excise the floxed G9a allele, myoblasts were treated with 1 µM 4-OHT (Sigma) for 48 h. The excision was confirmed by PCR. Primer sequences and PCR conditions are available upon request. Mouse C2C12 cells were cultured in Dulbecco’s modified Eagle's medium (DMEM) (Sigma) containing 20% fetal bovine serum (Hyclone). Cells were maintained at 60% confluency. To induce differentiation, 80–90% confluent cells were cultured in differentiation media (DMEM with 2% horse serum). The 293T cells and Phoenix cells were cultured in DMEM containing 10% FBS. COS-7 cells were cultured in DMEM with 10% calf serum (Hyclone).

### Proliferation and differentiation assays

Cellular proliferation was assessed using the BrdU incorporation assay kit (Roche) as described^22^. Proliferating cells were pulsed with 10 μM BrdU for 30 min. After fixation, cells were stained with anti-BrdU antibody. For cell cycle analysis of synchronized cells, proliferating cells were synchronized at the G_1_/S boundary using 1 mM hydroxyureaHU for 14 h and released in the growth media^22^. Cells were collected at different time points and stained with propidium iodide (10 μg/ml propidium iodide with 200 μg/ml RNase) for 30 min at room temperature. Cell cycle profiling was done with at least 15,000 cells using a flow cytometer (Becton Dickinson). Data were analyzed with the FlowJo software. To assess differentiation, cells cultured in differentiation medium were washed with phosphate-buffered saline (PBS) and fixed in 4% paraformaldehyde. After permeabilization, cells were stained with anti-MHC antibody (MY32; Sigma) followed by secondary antibody conjugated to Alexa Fluor (Molecular Probes). Slides were mounted in 4′,6-diamidino-2-phenylindole (DAPI) containing Vectashield mounting medium (Vector Laboratories). Images of cells in at least five random fields were captured using the Olympus (DP72) microscope. Differentiation was quantified by calculation myogenic index obtained by calculating the ratio of nuclei within the myotubes to the total number of nuclei. A minimum of 750 nuclei from at least five different fields were counted for each sample.

### Plasmids, retroviral infection, and siRNA knockdown

SUMO1 and SENP1 were kindly provided by Dr. Martin Lee^50^. pBABE, pBABE-G9a and Flag-G9a, and EGFP-G9a were a gift from Dr. Martin J. Walsh^51^. The QuikChange™ site-directed mutagenesis kit (Agilent) was used to mutate potential SUMOylation residues from lysine to arginine in Flag-G9a. Primers for generating the single mutants K79R, K125R, K256R, and K799R are provided in Table 1. The sites were sequentially mutated to generate Flag-G9a4KR, where all four acceptor lysines were changed to arginine. Primers for generating the same mutations in pBABE retroviral vector [pBABE-G9aK79R, pBABE-G9aK152R, pBABE-G9aK256R, pBABE-G9aK799R] are also listed in Table 1. All mutations were confirmed by sequencing the entire complementary DNA (cDNA). To generate stably overexpressing cell lines, Phoenix cells were transfected with pBABE, pBABE-G9a, or pBABE-G9a4KR using the calcium phosphate transfection kit (Invitrogen). The retroviral supernatant was used to infect C2C12 at 20–30% confluency. Thirty six hours post infection, cells were selected with 2 μg/ml puromycin (Sigma), amplified, and used for experiments. Flag-PCAF was provided by Dr.Yoshihiro Nakatani^52^. The QuikChange™ site-directed mutagenesis kit (Agilent) was used to mutate the hydrophobic residues in the SUMO-interacting motif Q**-**I**IV-**S**-L** to alanine. Mutations were confirmed by sequencing. Primers are provided in Table 1. To perform G9a-knockdown cells were transfected with 50 nM siRNA specific for G9a (ON-TARGET plus smart pool, Mouse BAT 8; accession number: NM 147151; NM 145830) from Dharmacon using Lipofectamine RNAiMAX (Invitrogen) according to the manufacturer’s instructions. Control cells were transfected with scrsiRNA (on-target plus control pool). Cells were analyzed 48 h after transfection of siRNA.

**Table 1:** List of primers to generate G9a SUMOylation mutants and PCAF-SIM mutant by site-directed mutagenesis

Primer	Sequence
pBABE-G9aK79R F	5′-GTGGACTCCGACAGCAGGTCTGAAGTTGAAGCT-3′
pBABE-G9aK79R R	5′-AGCTTCAACTTCAGACCTGCTGTCGGAGTCCAC-3′
pBABE-G9aK152R F	5′-CTCGGAAACGGCGCAGGCGGGAGC-3′
pBABE-G9aK152R R	5′-GCTCCCGCCTGCGCCGTTTCCGAG-3′
pBABE-G9aK256R F	5′-CAATGCCGCCATCCTCAGGCGGGAGAC-3′
pBABE-G9aK256R R	5′-GTCTCCCGCCTGAGGATGGCGGCATTG-3′
pBABE-G9aK799R F	5′-ATTGCTCCAGGAATTTAACAGGATTGAGCCTCCGCT-3′
pBABE-G9aK799R R	5′-AGCGGAGGCTCAATCCTGTTAAATTCCTGGAGCAAT-3′
Flag-G9aK79R F	5′-GACTCCGACAGCGGTCTGAAGTTGAA-3′
Flag-G9aK79R R	5′-TTCAACTTCAGA CCTGCTGTCGGAGTC-3′
Flag-G9aK152R F	5′-CGGAAACGGCGCAGGCGGGGA GCCTCCG-3′
Flag-G9aK152R R	5′-CGGAGGCTCCCGCCTGCGCCGTTTCCG-3′
Flag-G9aK256R F	5′- GCCGCCATCCTCAGGCGGGAGACCATG-3′
Flag-G9aK256R R	5′-CATGGTCTCCCGCTTGAGGATGGCGGC-3′
Flag-G9aK799R F	5′-CAGGAATTTAACAGGATTGAGCCTCCG-3′
Flag-G9aK799R R	5′-CGGAGGCTCAATCTTGTTAAATTCCTG-3′
PCAF SIM mutant F	5′-CCAGAGCCGACCTGCAGCAAATAGCTGCCAGTGCAACAGAATCCTGTCGGAG-3'
PCAF SIM mutant R	5′-CTCCGACAGGATTCTGTTGCACTGGCAGCTATTTGCTGCAGGTCGGCTCTGG-3′

*PCAF* P300/CBP-associated factor, *SIM* SUMO interaction motif, *F* forward, *R* reverse

### Protein SUMOylation

SUMOylation of wild-type G9a and single mutant proteins was assessed by the Global Protein SUMOylation assay kit (Abcam, ab115131). Briefly, proliferating C2C12 cells were co-transfected with the Flag-tagged wild-type G9a or mutants K79R, K152R, K256R, and K799R along with SUMO1. Control cells were transfected with SUMO1 and the empty vector pCS2. Twenty four hours post transfection, nuclear extracts were prepared using the nuclear extraction kit (Abcam, ab113474). Sample wells were incubated with anti-Flag antibody (Sigma) in binding buffer. After blocking and washes, samples were incubated in the SUMO assay buffer for 1 h. After incubation with the SUMO antibody, the detection solution and color development solution were added to the wells until a brilliant blue color was observed in the positive control wells. Negative control wells were colorless or light blue. Absorbance at 450 nm was recorded with the Varioskan plate reader using the SkanIt software. The absorbance of the negative control was subtracted from each sample. The absorbance for wild-type G9a was given an arbitrary value of 100% and the mutants were plotted relative to it.

### Reporter assays

The MyoD promoter reporter (4RTk-Luc) and the Cyclin D1 promoter reporter (pD1Luc) have been previously described^33,53^. pBABE, pBABE-G9a, and pBABE-G9a4KR cells were transfected with 200 ng of pD1Luc or 50 ng 4RTk-Luc reporters in 24-well plates. Five nanograms of Renilla reporter was co-transfected as a normalization control. Transfection was carried out in triplicates using Lipofectamine Plus. Reporter activity was analyzed with the Promega Dual-Luciferase® Reporter Assay System. Luminescence was analyzed with Varioskan plate reader using the SkanIt software.

### Subcellular localization

To examine subcellular localization of wild-type and G9a SUMOylation mutants, COS-7 cells were seeded at a density of 1 × 10^4^ cells/well in 6-well plates. After 24 h, cells were transfected with either Flag-G9a, single mutants or Flag-G9a4KR along with SUMO1. Cells were fixed after 48 h of transfection and incubated with mouse anti-Flag antibody (Sigma) and detected with Texas Red-coupled secondary antibody. Cells were visualized on a Zeiss LSM 510 META confocal laser scanning microscope.

### Immunoprecipitation and ChIP

To detect G9a SUMOylation, 293T cells were transfected with Flag-G9a or Flag-G9a4KR and SUMO1 expression vectors. Forty eight hours post transfection, cells were lysed in lysis buffer [50 mM Tris-HCl, pH 8.0, 50 mM NaCl, 1 mM EDTA, 0.1% Triton X-100, 0.5 mM phenylmethylsulfonyl fluoride, and protease inhibitors (Roche) containing 20 mM *N*-ethylmaleimide (Sigma)]. Lysates were immunoprecipitated using anti-Flag beads (Sigma) and immunoprecipitates were run on sodium dodecyl sulfate-polyacrylamide gel electrophoresis (SDS-PAGE) gels followed by western blotting. The blots were probed with anti-SUMO1 (Thermo Fisher Scientific), anti-FLAG, and anti-β-actin (Sigma) antibodies. For detecting endogenous SUMOylation, C2C12 lysates were immunoprecipitated using anti-G9a antibody (Millipore) and protein A/G beads (SC-2003), followed by immunoblotting and detection with anti-SUMO1 and anti-G9a antibodies (Cell Signaling). HEK cells were co-transfected with E2F1 and Flag-G9a or Flag-G9a4KR. After 48 h, cells were lysed in RIPA buffer (150 mM sodium chloride, 1% NP-40, 0.1% sodium deoxycholate, 50 mM Tris, pH 8.0, and 5 mM ethylenediaminetetraaceticacid with protease inhibitor). Lysates were immunoprecipitated with anti-Flag beads (Sigma) and subsequently run on SDS-PAGE gels followed by immunoblotting with anti-E2F1 (Santa Cruz) and anti-G9a antibodies (Cell Signaling). HEK cells were co-transfected with Flag-PCAF and Flag-G9a or Flag-G9a4KR. Forty eight hours after transfection, lysates were collected in RIPA buffer. Lysates were immunoprecipitated with anti-PCAF antibody (Santa Cruz). Protein A/G beads were used to pull-down PCAF. The immunoprecipitated material was run on SDS-PAGE gels followed by immunoblotting with anti-PCAF antibody (Santa Cruz) or anti-G9a antibody (Cell Signaling). For co-immunoprecipitation, C2C12 cells were transfected with pCS2 Flag-G9a or pCS2 Flag-G9a4KR. Twenty four hours post transfection, cells were lysed in NP-40 buffer (20 mM Tris-HCl, pH 7.4, 137 mM NaCl, 2 mM ethylenediaminetetraacetic acid, and 1% NP-40, 20 mM *N*-ethylmaleimide with protease inhibitor). The lysates were immunoprecipitated with anti-Flag-agarose beads (Sigma) or anti-E2F1 antibody (Abcam). Protein A/G agarose beads were used to pull-down E2F1. The immunoprecipitate was washed and loaded onto SDS-PAGE gels, followed by immunoblotting with anti-E2F1 (Abcam), anti-Flag (Sigma), anti-PCAF (Santa Cruz), and anti-β-actin (Sigma) antibodies. The following antibodies used for western blotting: anti-G9a (3306S), anti-Cyclin D1 (sc-753), anti-Cyclin A (sc-239), anti-p21 (sc-397), anti-MyoD (M-318) (sc-760), anti-Myogenin (sc-576), normal rabbit IgG (sc-2027), anti-PCAF (sc-13124), anti-E2F1 (ab179445), anti-E2F1 (sc-251), anti-Troponin-T (T6277), anti-Flag (F3165), and anti-β-actin (A1978). ChIP was carried out as described^22^ using the Millipore kit (17-295). Briefly, 10^6^ cells were crosslinked using 1% formaldehyde for 10 min at 37 °C. Cells were rinsed with PBS, lysed in SDS buffer, and sonicated using Bioruptor (Diagenode). Ten percent of sample was kept aside as input. Two micrograms of E2F1, PCAF, H3K9ac, H3K9me2, and Flag antibodies were used per immunoprecipitation. After washes, and reverse crosslinking, samples were treated with proteinase K and DNA was isolated using phenol–chloroform–isoamyl alcohol (Sigma). Q-PCR was performed to check relative enrichment on Cyclin D1 and DHFR promoters. Primers for Cyclin D1, DHFR, Myogenin, p21, and β-actin promoters for ChIP-PCRs have been previously described^22^. Q-PCR for each gene was performed in triplicates and are representative of at least two biological replicates. For western blots, bands intensities were quantified using the ImageJ software.

### Statistical analysis

*P* values were determined using Student’s *t* test and presented as mean ± SD (*p<0.05, **p<0.01, ***p<0.001).

## References

[1] Csizmok V, Forman-Kay JD (2018). Complex regulatory mechanisms mediated by the interplay of multiple post-translational modifications. Curr. Opin. Struct. Biol..

[2] Hilgarth RS (2004). Regulation and function of SUMO modification. J. Biol. Chem..

[3] Hay RT (2005). SUMO: a history of modification. Mol. Cell.

[4] Nayak, A. & Müller S. SUMO-specific proteases/isopeptidases: SENPs and beyond. *Genome Biol.* 1**5**, 10.1186/s13059-014-0422-2 (2014). 10.1186/s13059-014-0422-2 PMC4281951 25315341

[5] Verger A, Perdomo J, Crossley M (2003). Modification with SUMO. A role in transcriptional regulation. EMBO Rep..

[6] Hecker CM, Rabiller M, Haglund K, Bayer P, Dikic I (2006). Specification of SUMO1- and SUMO2-interacting motifs. J. Biol. Chem..

[7] Ouyang J, Gill G (2009). SUMO engages multiple corepressors to regulate chromatin structure and transcription. Epigenetics.

[8] Gareau JR, Lima CD (2010). The SUMO pathway: emerging mechanisms that shape specificity, conjugation and recognition. Nat. Rev. Mol. Cell. Biol..

[9] Flotho A, Melchior F (2013). Sumoylation: a regulatory protein modification in health and disease. Annu. Rev. Biochem..

[10] Eifler K, Vertegaal ACO (2015). SUMOylation-mediated regulation of cell cycle progression and cancer. Trends Biochem. Sci..

[11] Deyrieux, A. F. & Wilson, V. G. in *SUMO Regulation of Cellular Processes*197–214 (Springer, Cham, Switzerland, 2017).

[12] Garcia-Dominguez M, Reyes JC (2009). SUMO association with repressor complexes, emerging routes for transcriptional control. Biochim. Biophys. Acta.

[13] Morris JR, Garvin AJ (2017). SUMO in the DNA double-stranded break response: similarities, differences, and cooperation with ubiquitin. J. Mol. Biol..

[14] Shankar SR (2013). G9a, a multipotent regulator of gene expression. Epigenetics.

[15] Shinkai Y, Tachibana M (2011). H3K9 methyltransferase G9a and the related molecule GLP. Genes Dev..

[16] Tachibana M (2005). Histone methyltransferases G9a and GLP form heteromeric complexes and are both crucial for methylation of euchromatin at H3-K9. Genes Dev..

[17] Ling BM (2012). Lysine methyltransferase G9a methylates the transcription factor MyoD and regulates skeletal muscle differentiation. Proc. Natl Acad. Sci. USA.

[18] Wang J, Abate-Shen C (2012). The MSX1 homeoprotein recruits G9a methyltransferase to repressed target genes in myoblast cells. PLoS ONE.

[19] Choi J (2014). Modulation of lysine methylation in myocyte enhancer factor 2 during skeletal muscle cell differentiation. Nucleic Acids Res..

[20] Jung ES (2015). Jmjd2C increases MyoD transcriptional activity through inhibiting G9a-dependent MyoD degradation. Biochim. Biophys. Acta.

[21] Battisti V (2016). Unexpected distinct roles of the related histone H3 Lysine 9 methyltransferases G9a and G9a-like protein in myoblasts. J. Mol. Biol..

[22] Rao VK (2016). G9a promotes proliferation and inhibits cell cycle exit during myogenic differentiation. Nucleic Acids Res..

[23] Grégoire S (2006). Control of MEF2 transcriptional activity by coordinated phosphorylation and sumoylation. J. Biol. Chem..

[24] Riquelme C, Barthel KKB, Liu X (2006). SUMO-1 modification of MEF2A regulates its transcriptional activity. J. Cell. Mol. Med..

[25] Luan Z, Liu Y, Stuhlmiller TJ, Marquez J, García-Castro MI (2013). SUMOylation of Pax7 is essential for neural crest and muscle development. Cell Mol. Life Sci..

[26] Riquelme C, Barthel KKB, Qin XF, Liu X (2006). Ubc9 expression is essential for myotube formation in C2C12. Exp. Cell Res..

[27] Lee DY, Northrop JP, Kuo MH, Stallcup MR (2006). Histone H3 lysine 9 methyltransferase G9a is a transcriptional coactivator for nuclear receptors. J. Biol. Chem..

[28] Chaturvedi CP (2009). Dual role for the methyltransferase G9a in the maintenance of beta-globin gene transcription in adult erythroid cells. Proc. Natl Acad. Sci. USA.

[29] Purcell DJ, Jeong KW, Bittencourt D, Gerke DS, Stallcup MR (2011). A distinct mechanism for coactivator versus corepressor function by histone methyltransferase G9a in transcriptional regulation. J. Biol. Chem..

[30] Bittencourt D (2012). G9a functions as a molecular scaffold for assembly of transcriptional coactivators on a subset of glucocorticoid receptor target genes. Proc. Natl Acad. Sci. USA.

[31] Zhang X (2016). G9a-mediated methylation of ERα links the PHF20/MOF histone acetyltransferase complex to hormonal gene expression. Nat. Commun..

[32] Poulard C (2017). A post-translational modification switch controls coactivator function of histone methyltransferases G9a and GLP. EMBO Rep..

[33] Weintraub H, Davis R, Lockshon D, Lassar A (1990). MyoD binds cooperatively to two sites in a target enhancer sequence: occupancy of two sites is required for activation. Proc. Natl Acad. Sci. USA.

[34] Martínez-Balbás MA, Bauer UM, Nielsen SJ, Brehm A, Kouzarides T (2000). Regulation of E2F1 activity by acetylation. EMBO J..

[35] Kim KB (2015). H3K9 methyltransferase G9a negatively regulates UHRF1 transcription during leukemia cell differentiation. Nucleic Acids Res..

[36] Shiio Y, Eisenman RN (2003). Histone sumoylation is associated with transcriptional repression. Proc. Natl Acad. Sci. USA.

[37] Rosonina E, Duncan SM, Manley JL (2010). SUMO functions in constitutive transcription and during activation of inducible genes in yeast. Genes Dev..

[38] Dhall, A. et al. Sumoylated human histone H4 prevents chromatin compaction by inhibiting long-range internucleosomal interactions. *J. Biol. Chem*. jbc.M114.591644 (2014). 10.1074/jbc.M114.591644 PMC4256319 25294883

[39] Rodriguez MS (1999). SUMO-1 modification activates the transcriptional response of p53. EMBO J..

[40] Yamamoto H, Ihara M, Matsuura Y, Kikuchi A (2003). Sumoylation is involved in β-catenin-dependent activation of Tcf-4. EMBO J..

[41] Gómez-del Arco P, Koipally J, Georgopoulos K (2005). Ikaros SUMOylation: switching out of repression. Mol. Cell. Biol..

[42] Schimmel J (2014). Uncovering SUMOylation dynamics during cell-cycle progression reveals FoxM1 as a key mitotic SUMO target protein. Mol. Cell.

[43] Ling Y (2004). Modification of de novo DNA methyltransferase 3a (Dnmt3a) by SUMO-1 modulates its interaction with histone deacetylases (HDACs) and its capacity to repress transcription. Nucleic Acids Res.

[44] Schaefer A (2009). Control of cognition and adaptive behavior by the GLP/G9a epigenetic suppressor complex. Neuron.

[45] Zhang RH, Judson RN, Liu DY, Kast J, Rossi FMV (2016). The lysine methyltransferase Ehmt2/G9a is dispensable for skeletal muscle development and regeneration. Skelet. Muscle.

[46] Casciello F (2017). G9a drives hypoxia-mediated gene repression for breast cancer cell survival and tumorigenesis. Proc. Natl Acad. Sci. USA.

[47] Seeler JS, Dejean A (2017). SUMO and the robustness of cancer. Nat. Rev. Cancer.

[48] Rando TA, Blau HM (1994). Primary mouse myoblast purification, characterization, and transplantation for cell-mediated gene therapy. J. Cell Biol..

[49] Sun, H. et al. Stra13 regulates satellite cell activation by antagonizing Notch signaling. *J. Cell Biol.* **177**, 647–657 (2007). 10.1083/jcb.200609007 PMC2064210 17502421

[50] Wang Y, Shankar SR, Kher D, Ling BMT, Taneja R (2013). Sumoylation of the basic helix–loop–helix transcription factor sharp-1 regulates recruitment of the histone methyltransferase G9a and function in myogenesis. J. Biol. Chem..

[51] Nishio H, Walsh MJ (2004). CCAAT displacement protein/cut homolog recruits G9a histone lysine methyltransferase to repress transcription. Proc. Natl Acad. Sci. USA.

[52] Yang XJ, Ogryzko VV, Nishikawa J, Howard BH, Nakatani Y (1996). A p300/CBP-associated factor that competes with the adenoviral oncoprotein E1A. Nature.

[53] Hinz M (1999). NF-kappaB function in growth control: regulation of cyclin D1 expression and G0/G1-to-S-phase transition. Mol. Cell. Biol..

